# Quantifying the human visual exposome with vision language models

**DOI:** 10.1038/s44488-026-00023-x

**Published:** 2026-11-05

**Authors:** Christian Rominger, Andreas R. Schwerdtfeger, Malay Gaherwar Singh, Dimitri Khudyakow, Elizabeth A. M. Michels, Fabian Wolf, Jakob Nikolas Kather, Magdalena Katharina Wekenborg

**Affiliations:** 1 Health Psychology, University of Graz, Graz, Austria; 2 Else Kroener Fresenius Center for Digital Health, Faculty of Medicine and University Hospital Carl Gustav Carus, TUD Dresden University of Technology, Dresden, Germany; 3 Department of Medicine I, Faculty of Medicine and University Hospital Carl Gustav Carus, TUD Dresden University of Technology, Dresden, Germany; 4 Medical Oncology, National Center for Tumor Diseases (NCT), University Hospital Heidelberg, Heidelberg, Germany

**Keywords:** Environmental sciences, Psychology, Psychology

## Abstract

The visual environment is a fundamental yet unquantified determinant of mental health. While the concept of the environmental exposome is well-established, current methods rely on coarse geospatial proxies or biased self-reports, failing to capture the first-person visual context of daily life. Here we show that coupling ecological momentary assessment with vision-language models can quantify the semantic richness of human visual experience. Across 2,674 participant-generated photographs, model-derived estimates of “greenness” robustly predicted momentary affect and chronic stress, consistent with established benchmarks. We then developed a semi-autonomous large language model-based pipeline that mined over seven million scientific publications to extract nearly 1,000 environmental features empirically linked to mental health. When applied to real-world imagery, up to 33% of model-extracted context ratings significantly correlated with affect and stress.

## Introduction

Mental illness creates an immense global burden on public health and economies^1^. Decades of research suggest that everyday surroundings exert a profound influence on mental health^2^, with greenness emerging as one of the most consistently replicated environmental predictors^3–6^. Collectively, these surroundings can be summarised as the 'environmental exposome'^7^, with the visual perception of the environment termed the visual exposome. Still, our ability to understand and quantify how the visual exposome shapes mental health remains surprisingly limited: we can measure where people are and how they subjectively perceive their surroundings, but we remain effectively blind to what people actually see in their daily lives. GPS-based metrics and satellite-derived indices provide useful macro-level proxies of environmental features^4,8–10^, but they cannot cover the semantic richness, spatial nuance, or social signals embedded in real-world visual scenes. Most crucially, they overlook the immediate, first-person environments individuals actually encounter, whether a space is crowded or quiet, whether animals or other people are present, whether natural light, greenery, or stress-inducing cues appear in view^11,12^. Self-report measures reflect only subjective impressions and are prone to response biases. As a result, the visual exposome, arguably the most proximal form of contextual exposure, has remained empirically inaccessible at scale.

An optimal way to quantify the visual exposome would be to use photographs taken by participants themselves to capture health-relevant contextual features of their environments. This method provides a large number of data points that reflect the participant’s surroundings from a first-person perspective, allowing researchers to see the world through the participants’ eyes. To date, however, this approach has been used almost exclusively in qualitative research, for example, to explore subjective experiences, health contexts, or to illustrate environmental features^13–15^, but not in quantitative frameworks examining associations between visually captured aspects of daily environments and psychological and mental health-relevant outcomes. This limited application is most likely due to the reliance on manual expert evaluation of visual content according to predefined categories, an approach that is time-consuming, subjective and inherently not scalable, restricting its use in intensive longitudinal studies with large sample sizes. Recent advances in vision-language models (VLMs) now allow us to automatically extract semantically meaningful features from images, as illustrated by their near-human-level scene understanding across domains such as autonomous driving^16,17^.

Crucially, unlike conventional, single-purpose computer-vision models that require task-specific training for each target feature, VLMs can rate arbitrary features without retraining, allowing a single pipeline to quantify a virtually unlimited range of contextual features from the same images. Therefore, we hypothesised that VLMs could serve as scalable, objective tools to decode the complex visual information encoded in photographs. Embedded within an ecological momentary assessment framework^18^, this could effectively unlock a new dimension of human phenotyping: continuous, first-person mapping of the visual exposome in its entirety.

As a first step, we applied VLMs to well-established greenness-related features (i.e. *greenness, nature score*, *plant presence, natural light exposure* and *inside/outside*)^12,19,20^, using them as a benchmark to assess whether VLM-derived estimates from 2674 participant-generated photographs correspond to participants’ self-ratings of greenness and capture the well-documented associations with mental health-relevant outcomes reported in prior work^2,8,20^.

Next, we mapped the entirety of environmental features previously linked to affect and stress. We developed and applied a semi-autonomous AI-based literature mining system that analyzed more than seven million scientific publications to identify nearly 1000 contextual features with empirical support for associations with affect and stress.

These features were then used as objectives for VLM-based ratings, allowing us to examine whether the resulting VLM-derived contextual features can reproduce the empirically well-documented associations of the human exposome. We find that model-derived greenness estimates reproduce established associations with affect and chronic stress, and that a substantial proportion of the nearly 1000 literature-derived features show the expected associations in real-world imagery, demonstrating that the human visual environment can be quantified and mapped to mental health-relevant outcomes at scale.

## Results

### Participant characteristics and study design

**Fig. 1:**
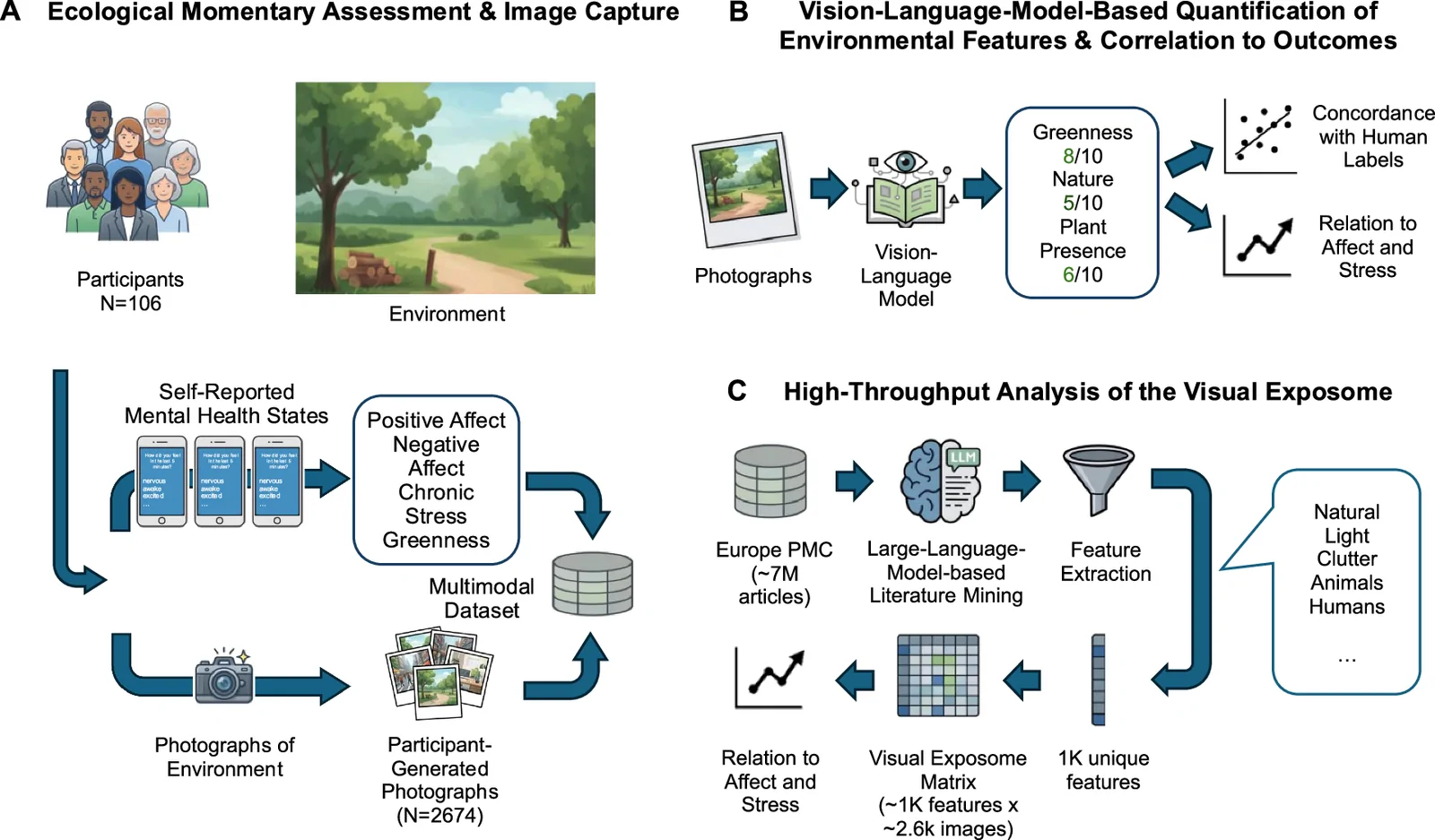
Framework for first-person visual exposomics: data collection, model validation and large-scale feature discovery. **A** Study design and real-world data acquisition. Participants completed a 7-day ambulatory protocol, capturing 2674 photographs that reflect their moment-to-moment visual environments alongside repeated assessments of affect and stress. **B** Validation of vision-language model (VLM)-derived contextual quantification. Across 2674 participant-generated photographs, VLM-based estimates of greenness robustly predicted momentary affect and chronic stress, replicating established associations from environmental psychology and demonstrating the validity of VLM-derived contextual measures. **C** Large-scale contextual feature discovery and application. A semi-autonomous large language model (LLM)-driven system mined >7 million scientific publications, identifying nearly 1000 environmental features previously linked to mental health. When applied to real-world imagery, up to 33% of VLM-extracted contextual ratings showed significant associations with affect and stress, highlighting the scalability and breadth of VLM-based visual exposomics.

We conducted an ecological momentary assessment study combining self-reports, participant-generated photographs, and an AI-driven literature-mining approach to test whether VLMs can quantify a wide range of real-world environmental features relevant to mental health (Fig. 1). Data from 106 adults (28 men; mean age = 24.9 ± 10.7 years) who participated in a 7-day ecological momentary assessment study using the *ESMira* smartphone application were available^21^. Participants received seven randomly timed alarms per day between 09:00 a.m. and 08:00 p.m. to complete brief self-reports on indicators of mental health, namely positive and negative affect, and contextual information on greenness while simultaneously capturing a photograph of their current environment from their own perspective. In total, 2674 participant-generated photographs in combination with self-reports were available for analysis. Participants further completed a questionnaire on sociodemographic variables and chronic stress. All subsequent analyses are based on this dataset. A detailed description of our methodology is provided in the 'Methods'.

### VLMs quantify greenness and related visual features linked to mental health

As an initial step to validate the VLM framework against established evidence, we focused on greenness as a core visual property with well-established links to mental health. Beyond greenness, we quantified four visual environmental features that commonly accompany it, namely, *plant presence*, *nature score* (degree of natural scenery), *natural light exposure* (the intensity of daylight) and *inside/outside* classification, yielding five greenness-related visual measures derived from the VLMs. Each participant-generated photograph was subsequently rated 5 times on these dimensions by the open-weight LLaMA 4 VLM, which also provided a confidence score for every rating (see Supplementary Table 1 for more details). We then validated these model-derived ratings by examining their correspondence with participants’ subjective experience of their surroundings. Multilevel analysis revealed significant associations between participants’ self-reported greenness ratings and VLM-derived ratings of greenness itself and related visual features. More precisely, participants rated their surroundings as greener when their photographs were characterised by higher VLM-derived ratings of greenness (*t*(2571.73) = 44.41, *b* = 1.26, *p* < 0.001) and the other greenness-related features (i.e. *plant presence*, *natural light exposure* and *inside/outside;* see Table 1).

**Table 1:** Multilevel model predicting objective indicators of greenness via subjective greenness ratings

Predictors	Greenness			Natural light exposure			Plant presence			Nature score			Inside/outside			Average score		
	Estimates	Conf. Int. (95%)	*p*-value	Estimates	Conf. Int. (95%)	*p*-value	Estimates	Conf. Int. (95%)	*p*-value	Estimates	Conf. Int. (95%)	*p*-value	Estimates	Conf. Int. (95%)	*p*-value	Estimates	Conf. Int. (95%)	*p*-value
Intercept	−0.04	−0.65 to 0.57	0.898	3.58	2.92–4.25	<0.001	0.11	−0.64 to 0.86	0.774	0.03	−0.53 to 0.60	0.913	1.05	0.97– 1.12	<0.001	0.93	0.34– 1.51	0.002
Subjective greenness trait (Level 2)	1.28	1.05–1.51	<0.001	0.52	0.27–0.77	<0.001	1.35	1.07– 1.63	<0.001	1.09	0.88– 1.31	<0.001	0.08	0.05– 0.11	<0.001	1.06	0.84– 1.28	<0.001
Subjective greenness state (Level 1)	1.26	1.20– 1.31	<0.001	0.75	0.66–0.83	<0.001	1.38	1.31– 1.44	<0.001	1.03	0.97– 1.08	<0.001	0.14	0.13– 0.15	<0.001	1.10	1.05– 1.15	<0.001

Statistical significance for fixed effects was determined using two-sided Wald t-tests within a linear mixed-effects framework. Exact *p*-values are provided for all fixed effects in the table. No adjustments were made for multiple comparisons. Level 1 variables represent state-level observations; Level 2 variables represent participant-level traits. Confidence intervals (Conf. Int.) are reported at the 95% level.

To assess whether these VLM-derived environmental features capture established relationships between greenness and mental health-related variables, we examined their associations with both momentary state-level (i.e. short-term experiences, namely positive and negative affect) and trait-level measures (more stable characteristics, namely mean affect over the study period and chronic stress). Across both time scales, VLM-derived visual greenness features were robustly associated with mental health-related variables (Table 2). Specifically, greater greenness was linked to higher momentary positive affect (*t*(2564.8) = 2.64, *b* = 0.20, *p* = 0.008).

**Table 2:** Multilevel model predicting VLM derived indicators of greenness via health-relevant variables

Predictors	Greenness			Natural light exposure			Plant presence			Nature score			Inside/outside			Average score		
	Estimates	Conf. Int. (95%)	*p*-value	Estimates	Conf. Int. (95%)	*p*-value	Estimates	Conf. Int. (95%)	*p*-value	Estimates	Conf. Int. (95%)	*p*-value	Estimates	Conf. Int. (95%)	*p*-value	Estimates	Conf. Int (95%)	*p*-value
Intercept	2.80	0.97–4.63	0.003	3.70	2.16–5.23	<0.001	2.79	0.67–4.92	0.010	2.07	0.46–3.68	0.012	1.27	1.09 – 1.45	<0.001	2.81	1.16–4.46	0.001
PA trait (Level 2)	0.62	0.15–1.09	0.010	0.46	0.07–0.85	0.021	0.66	0.11–1.21	0.018	0.62	0.21–1.04	0.003	0.02	−0.02 to 0.07	0.293	0.59	0.17–1.02	0.006
PA state (Level 1)	0.20	0.05–0.35	0.008	−0.03	−0.21 to 0.16	0.786	0.12	−0.06 to 0.29	0.189	0.06	−0.08 to 0.19	0.422	0.04	0.02–0.06	0.001	0.09	−0.05 to 0.22	0.206
NA trait (Level 2)	−1.07	−1.88 to −0.25	0.010	−0.10	−0.78 to 0.58	0.773	−0.90	−1.85 to 0.04	0.061	−0.82	−1.54 to −0.11	0.024	−0.07	−0.15 to 0.01	0.095	−0.70	−1.43 to 0.03	0.061
NA state (Level 1)	0.06	−0.19 to 0.31	0.620	−0.10	−0.40 to 0.20	0.529	0.04	−0.25 to 0.33	0.793	−0.01	−0.24 to 0.21	0.927	−0.00	−0.04 to 0.04	0.905	−0.00	−0.23 to 0.22	0.986

Statistical significance for fixed effects was determined using two-sided Wald t-tests within a linear mixed-effects framework. Exact *p*-values are provided for all fixed effects in the table. No adjustments were made for multiple comparisons. Level 1 variables represent state-level observations; Level 2 variables represent participant-level traits. Confidence intervals (Conf. Int.) are reported at the 95% level.

*PA* positive affect, *NA* negative affect.

**Fig. 2:**
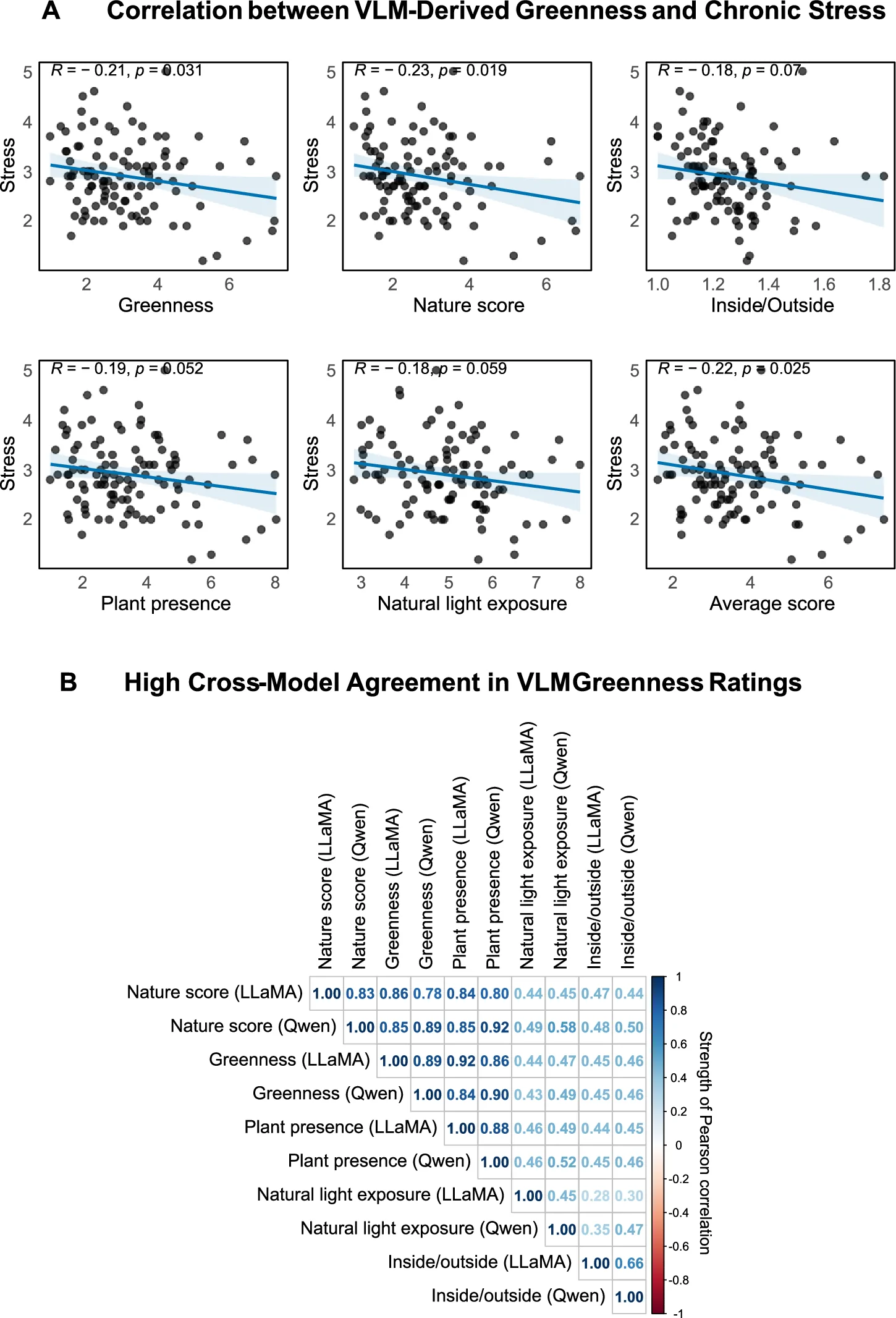
Greenness-stress associations and cross-VLM consistency in contextual ratings. **A** Trait-level associations between vision-language model (VLM)-derived greenness features and chronic stress. Higher greenness was significantly associated with lower chronic stress, with all greenness features showing negative correlations of comparable magnitude. The solid blue line represents the linear regression line of best fit, and the shaded error bands represent the 95% confidence intervals (CI). **B** Pearson correlations illustrating consistency of greenness-related feature ratings across two VLMs (LLaMA 4 VLM and open-weight Qwen3 VL). Both models produced highly similar ratings for greenness, nature score, and plant presence, with especially high consistency for nature score and greenness. The blue colour represents positive Pearson correlations, and red are negative Pearson correlations. Source data are provided in Supplementary Data 2.

At the trait level, mental health-related variables also showed systematic relationships with greenness-related features (e.g. *natural light exposure* and positive affect: *t*(105.6) = 2.31, *b* = 0.46, *p* = 0.022; for more details, see Table 2). Participants reporting higher average positive affect and lower negative affect over the study period tended to capture photographs containing more greenness, as reflected in higher *greenness* (positive affect: *t*(104.7) = 2.57, *b* = 0.62, *p* = 0.012, negative affect: *t*(109.7) = − 2.58, *b* = − 1.07, *p* = 0.011) and *nature score* (positive affect: *t*(103.3) = 2.96, *b* = 0.63, *p* = 0.004, negative affect: *t*(108.2) = − 2.25, *b* = − 0.82, *p* = 0.026) values. The average score of continuous VLM-derived greenness indicators was also significantly associated with higher trait positive affect (*t*(104.6) = 2.75, *b* = 0.62, *p* = 0.007). Being *inside/outside* (1 vs. 2) was linked with a higher positive affective state (*t*(2557.0) = 3.26, *b* = 0.04, *p* = 0.001). Similarly, higher VLM-derived *greenness* (*r* = −0.21, *p* = 0.028) and *nature score* (*r* = − 0.23, *p* = 0.019) were associated with lower perceived chronic stress at trait level. The other two VLM-derived greenness indicators, as well as *inside/outside*, showed negative effects of similar sizes (*rs* ≥ − 0.18; see Fig. 2), indicating comparable findings for all VLM-derived greenness indicators. The average score of continuous VLM-derived greenness indicators showed a significant correlation with *r* = − 0.22 (*p* = 0.025). Together, these data show that VLMs can reliably quantify health-relevant environmental features from real-world images and capture their meaningful associations with positive affect, negative affect, and stress.

When repeating these analyses using two simpler and computationally less demanding image-analysis methods, a green-pixel count and an object-detection approach (see Supplementary Methods), the overall pattern of findings was only partially reproduced. Specifically, the green-pixel measure showed a pattern of associations with positive and negative affect that was broadly comparable to the VLM-derived greenness ratings, whereas the object-detection-based plant-presence measure did not yield significant associations with affect (Supplementary Table 2). Neither approach recovered the association between visual environmental features and chronic stress that was observed for the VLM-derived measures (Supplementary Fig. 1). Together, these results suggest that VLM-derived ratings capture aspects of the visual environment that are not fully captured by simpler image-analysis approaches.

### VLMs yield consistent contextual ratings linked to mental health across models

To evaluate the generalizability of our findings across different model architectures, we compared the contextual feature ratings generated by LLaMA 4 VLM with those produced by the open-weight Qwen3 VL. As illustrated in Fig. 2B, both models yielded highly similar ratings for *greenness*, *nature score* and *plant presence* (*rs* = 0.83–0.89, all *ps* < 0.001). The correlation for *natural light exposure* (*r* = 0.45) and *inside/outside* was lower (*r* = 0.66; both with *p* < 0.001), suggesting slightly greater variability in this feature between models. As detailed in Supplementary Table 3, Qwen3 VL reproduced the overall pattern of associations between VLM-derived greenness-related features and mental health-relevant variables (i.e. state- and trait-level positive and negative affect) observed in the primary analyses with LLaMA 4 VLM (see Supplementary Table 3). Furthermore, *natural light exposure*, *plant presence*, and *greenness* showed a trend association with stress as a mental health-related variable, with similar effect sizes in the replication analyses (Supplementary Fig. 2). These results confirm that VLM-derived contextual ratings are consistent across models to effectively capture mental health-relevant environmental features.

### The VLM framework generalises to a broad literature-derived space of contextual features

**Fig. 3:**
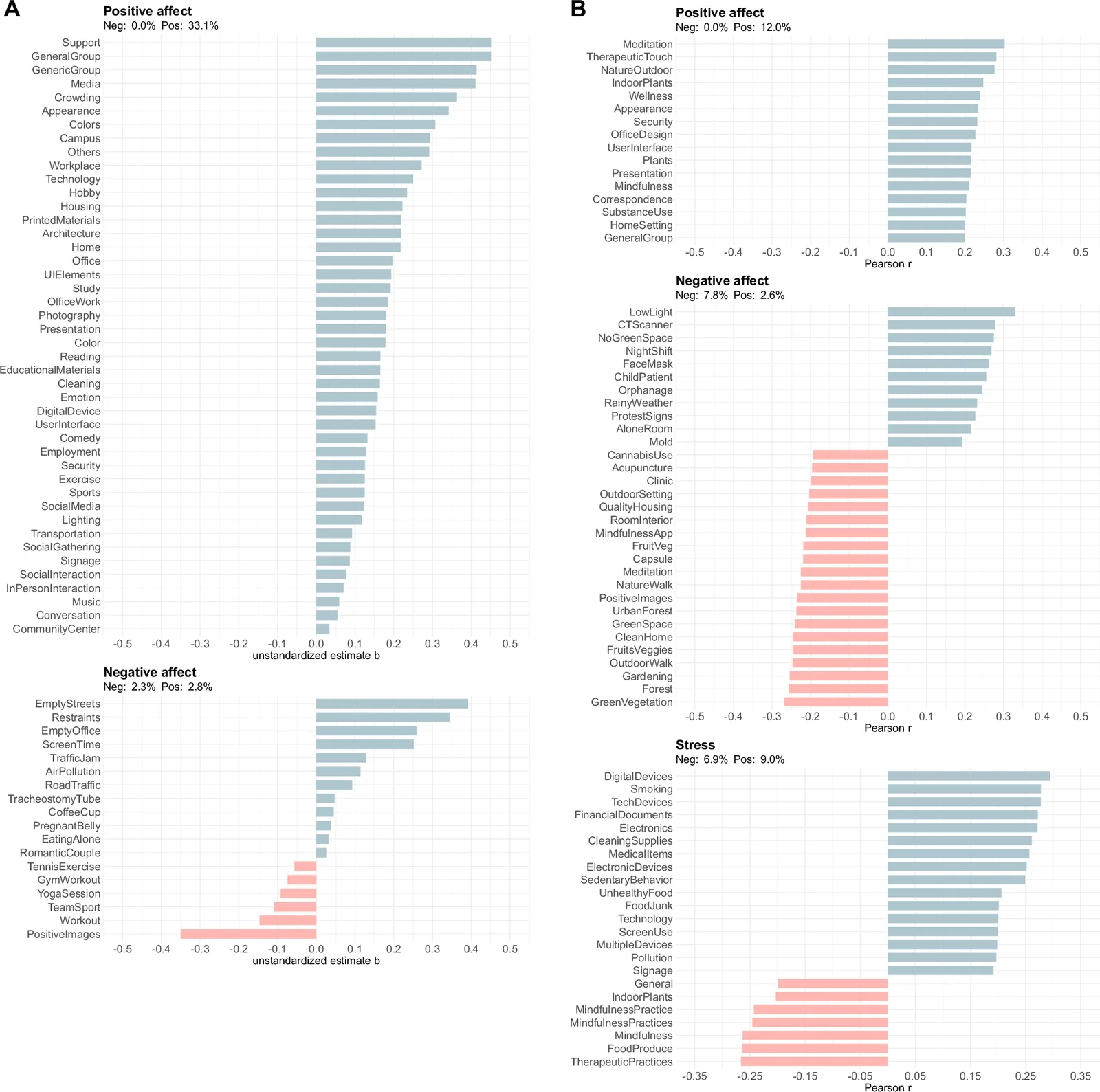
Associations between the literature-derived visual exposome, affect and stress. Significant associations were defined as *p* < 0.05. Positive associations are shown in blue, negative associations in pink. **A** Literature-derived visual exposome features associated with momentary affect and stress, where *b* stands for the unstandardised regression coefficient. **B** Literature-derived visual exposome features associated with enduring affect and stress, where Pearson *r* represents the strength of the relationship. Source data are provided in Supplementary Data 3.

To examine whether the framework extends beyond greenness-related features to a broader conceptual space of environmental determinants of mental health, we mapped environmental features previously linked to mental health using a large-scale, AI-driven literature mining approach. In a multiple-step process, we analyzed more than seven million open-access full-text articles from the Europe PMC database using the open-weight GPT-OSS-120B model, yielding a large set of 997 unique literature-derived contextual variables with empirical support for associations with affect and stress (prompts, code, and results are openly available; see Supplementary Fig. 3). These identified features were then used as prompts for VLM-based ratings of the participant-generated photographs, following the same procedure as for the greenness-related features. Across these features, VLM-derived ratings again showed significant associations with mental health-related variables, underscoring the broad applicability of the framework to identify mental health-relevant information in real-world environments (Fig. 3). For state variables of positive and negative affect, up to 33% of all context variables showed the expected direction of correlation at *p* < 0.05 (see Fig. 3A), whereas for trait measures, up to 12% showed the expected pattern (Fig. 3B). Together, these findings demonstrate that VLMs can accurately quantify a wide range of mental health-relevant contextual features in everyday photographs, enabling the scalability of image-based context mapping.

## Discussion

The environmental exposome is widely recognised as a fundamental determinant of human health, yet its immediate visual component has remained effectively unmeasured. While current epidemiological methods use geospatial proxies or self-report to estimate environmental exposure, these tools fail to capture the semantic content of the first-person human experience. Here, we define and quantify the visual exposome. By coupling ecological momentary assessment with VLMs, we resolve the measurement gap between macro-level environmental data and micro-level individual perception. Our results demonstrate that this framework allows for the scalable, objective decoding of associations between the visible world and affect and stress, both closely linked to mental health.

Our initial validation focused on greenness, one of the most consistently replicated environmental predictors of mental health^3^. We found that VLM-derived estimates of greenness and related features aligned robustly with participants’ subjective self-reports of their visual environment and showed the expected associations with state and trait affect, as well as chronic stress. This validates that VLMs can serve as an automated proxy for human environmental assessment. Unlike satellite-derived indices, which suffer from the 'uncertain geographic context problem' by capturing a location rather than the actual field of view, our approach directly evaluates what participants were visually exposed to. By analysing first-person images instead of geospatial proxies, this method provides a higher-resolution phenotype of environmental exposure that avoids the biases of self-report and the spatial coarseness of conventional metrics.

Beyond validating established associations, our findings reveal the immense complexity of the visual exposome. By developing and employing an AI-driven literature mining system to identify nearly one thousand distinct contextual features, we extended our analysis far beyond the traditional focus on greenness-associated features. The observation that a substantial proportion of these VLM-extracted context ratings significantly correlated with affective states suggests that mental health is shaped by a dense network of visual micro-exposures that have historically gone unmeasured. This represents a paradigm shift from hypothesis-driven studies of single environmental features^7^ toward a high-throughput approach capable of simultaneously quantifying thousands of visual determinants of health in real-world imagery. We emphasize that these analyses are confirmatory by design: because the features were selected on the basis of previously reported associations with affect and stress, our results test and illustrate the applicability of the VLM-based framework rather than discovering novel determinants.

This methodological advance addresses a critical scalability bottleneck in psychology and public health. Analyzing the semantic content of first-person images has traditionally required labour-intensive manual coding, restricting such data to qualitative studies or small samples. By automating this process with high consistency across different model architectures, we demonstrate that visual exposomics is viable for large-scale, intensive longitudinal cohorts. This capability parallels recent technological shifts, such as wearable biosensors enabling continuous physiological monitoring at scale^22,23^, and LLMs reshaping psychotherapy delivery, diabetes care^24^ and personalised health coaching^25^. Just as these earlier innovations, none originally designed for their current application, reshaped how entire fields could operate, VLMs now open a comparably far-reaching frontier. This highlights the great potential of this approach to further contribute to the field of precision public health by integrating detailed contextual information into personalised and individualised models^26^.

Our study has limitations. The cohort was exclusively European, limiting global generalizability, and the sample size precludes the detection of small effect sizes for rare environmental features. Screening hundreds of features is also computationally demanding (~67 h here), though targeted analyses of selected features run on standard research hardware (~1.6 h). However, we expect this barrier to diminish as inference efficiency improves and compute becomes more affordable.

Furthermore, the micro-longitudinal design captures associations rather than causality. For example, positive affect may drive individuals to seek visually enriching environments as much as the environment influences affect. Future work must leverage this VLM framework in diverse, cross-national cohorts and apply causal inference models to disentangle these bidirectional effects. Combining first-person imagery with ambulatory assessment makes such large-scale, cross-national studies both especially informative and increasingly feasible.

## Conclusions

This study provides evidence that the human visual environment can be objectively quantified and mapped to affect and stress, both central to mental health. By coupling ecological momentary assessment with VLMs, we close the measurement gap between macro-level environmental data and the first-person visual context individuals actually encounter. The framework is scalable, relies on openly available models that can be deployed locally, and generalises from greenness to a broad, literature-derived space of contextual features, providing a foundation for studying how the visible world shapes mental and physical diseases^11^.

## Methods

### Study approval and participants

Ethics approval was obtained from the Ethics Committee of the University of Graz (reference number: GZ. 39/168/63 ex 2024/25). The study was conducted in accordance with the ethical principles of the Declaration of Helsinki and institutional ethical standards. Participants were recruited via social media and personal contact. All participants provided informed consent before study enrollment and received course credits for participation.

### Ecological momentary assessment protocol

Of the 115 adults who participated, nine participants provided data from only one alarm. The remaining 106 adults (28 men), who completed the 7-day ecological momentary assessment via the *ESMira* smartphone application^21^, were included in all analyses. The app delivered seven random alarms per day between 09:00 a.m. and 08:00 p.m., with a minimum interval of 30 min between prompts. Participants could postpone alarms if necessary. At each alarm, they reported their momentary affective well-being and the perceived greenness of their surroundings. In addition, they were instructed to capture a photograph of their current environment from their own perspective. Participants could choose to skip taking a photograph if they preferred. In total, 2674 images with corresponding momentary self-reports were collected (i.e. *M* = 25.2 images per participant, SD = 9.96). Assessments began on a random weekday and continued for 7 consecutive days. At the start of the ecological momentary assessment period, participants completed baseline questionnaires covering demographic and health-relevant information, including age, sex and chronic stress.

### Mental health-related variables

We assessed momentary affect via 10 items, which were subdivided into positive affect (5 items: active, awake, stimulated, determined, attentive) and negative affect (5 items: angry, hostile, ashamed, nervous, anxious)^27^, each rated on a five-point Likert scale from 1 (not at all) to 5 (very much). Reliability analyses suggested good within-person and good between-person reliability for positive affect (*R*_Cn_ = 0.64, *R*_kRn_ = 0.97) and negative affect (*R*_Cn_ = 0.60, *R*_kRn_ = 0.97)^28^. Between-person variables (trait Level 2) were computed as each participant’s mean across all momentary assessments; within-person variables (state Level 1) were the affect ratings at each alarm. The mean positive affect over all alarms was *M* = 2.89 (SD = 0.84), and the mean negative affect was *M* = 1.31 (SD = 0.49).

Additionally, we assessed momentary perceived greenness with a single item ('How green do you perceive your current environment?') rated from *not at all green* to *extremely green* on a six-point Likert scale (*M* = 2.49, SD = 1.51).

In addition to these momentary assessments, we collected chronic stress using the Perceived Stress Scale (PSS)^29^. The PSS measures the subjective experience of stress during the past weeks^30^. We used the validated German version^31^ with ten items rated on a five-point Likert scale from 1 (never) to 5 (very often). Internal consistency was good (Cronbach’s *α* = 0.88). The mean score was *M* = 2.90 (SD = 0.72).

### Vision-language model analyses for greenness and related visual features

We primarily used the open-weight LLaMA 4 VLM (model: Maverick-17B-128E-Instruct-FP8; ≈17 billion active parameters; temperature = 0.6), selected as a state-of-the-art open-weight model that can be deployed locally without commercial API access, and accessed via a chat completion endpoint. Vision-language models were used as instruction-tuned models in a non-thinking (non-reasoning) configuration and were prompted to return each rating directly in the required structured format, without requesting chain-of-thought traces. The 'Instruct' designation of the Qwen3 VL model denotes the non-reasoning variant, as opposed to the separate 'Thinking' variant. No chain-of-thought prompting (e.g. 'think step by step') was applied, and no chain-of-thought traces were retained or parsed.

The model was prompted to generate quantitative ratings of greenness and visual features indicative of greenness in real-world environments, namely *greenness*, *nature score*, *plant presence* and *natural light exposure,* as well as a rating for *inside/outside* for each participant-generated photograph. To prevent potential cross-influences between categories, prompts were submitted individually, with five independent runs performed for each category. Mean scores across the five runs were used for subsequent analyses. Model outputs were parsed and stored in a structured format for subsequent statistical analysis. We conducted reliability analyses similar to those performed on affect, where runs served as items. We found high within-person (*R*_Cn_ = 0.97 –0.99) as well as between-person (*R*_kRn_ = 0.76–0.94) reliability across all runs. This argues for the calculation of mean scores for subsequent analyses. Detailed results for all five runs, including means and standard deviations, are provided in Supplementary Table 1. Each category was rated using a separate, single-category prompt of identical structure, in which only the category name was substituted. For the greenness-related features, the prompt was (shown here for ‘Greenness’):

*Here are the given categories: 'Greenness', 'GreennessConfidence', 'ImageDescription'. Analyze the image and evaluate accordingly from 1 to 10, whereas 1 is the absolute absence and 10 is abundance of the subject mentioned in criteria. For the category with Confidence in it give out a value from 1 to 10 of how confident you are about the evaluation of the category. For image description, describe what is to be seen in the picture. For output, use the following format: Categoryname: value, Category2name: value2. Do not say anything else*.

For the inside/outside classification, the rating instruction was replaced by 'use 1 for Inside and 2 for Outside'. Prompt design followed an iterative refinement process. An initial conversational, instruction-heavy prompt style proved inefficient: it increased prompt length and token cost, lengthened runtime, and reduced the model’s focus on task-relevant content.

We therefore adopted a deliberately concise, imperative prompting strategy based on established prompting practices, structured around five principles: (1) a brief statement of the task context, (2) a clear definition of the core rating task, (3) specification of a fixed output format, (4) an explicit instruction not to return additional content, and (5) concise, imperative phrasing. This design yielded consistent ratings in the required, machine-parseable format and was further refined over several minor iterations without subsequent substantive changes. The same prompting strategy was applied to the prompts used for the broader set of literature-derived features (see below).

To assess the generalizability of the approach beyond a single model architecture, the same procedure, including separate prompt submission, five independent runs per category, and averaging of resulting scores, we applied Qwen3 VL (model: Qwen3-VL-235B-A22B-Instruct-FP8; ≈22 billion active parameters; temperature = 0.7). We found similar high within-person (*R*_Cn_ =  0.99) as well as between-person reliability (*R*_kRn_ = 0.83–0.94) scores across all runs as for the LLaMA4 VLM ratings (see Supplementary Table 1). All models were executed in November 2025 on a locally hosted high-performance computing node equipped with eight Nvidia H200 GPUs and managed through the vLLM inference engine. Within the inference pipeline, each photograph was downscaled on the fly to a maximum long-edge resolution of 1280 pixels (preserving the original aspect ratio) using the Pillow library (version 12.1.0) in Python (version 3.13.2) with Lanczos resampling; the original full-resolution images (2674 photographs, ~37.71 GB) were retained on standard local storage, while the downscaled versions were generated only transiently for inference and not stored. The greenness analyses processed approximately 139.4 million input and 2.8 million output tokens with LLaMA 4, and 91.8 million input and 4.8 million output tokens with Qwen3 VL; the analysis of all 997 literature-derived features with LLaMA 4 processed approximately 5.57 billion input and 112.8 million output tokens.

As an indication of runtime on our hardware (without maximising concurrency, to preserve capacity for other workloads), cumulative inference time was approximately 1.6 h (LLaMA 4) and 2.5 h (Qwen3 VL) for the five-run greenness analyses, and approximately 67 h for the single-run analysis of all 997 literature-derived features.

### Vision-language model analyses beyond greenness

We next examined the generalizability of the framework beyond greenness, focusing on an extensive set of contextual features that are empirically linked with mental health-related variables (positive affect, negative affect and stress) and are potentially identifiable by VLMs in participant-generated photographs. To systematically map this conceptual space, we conducted an AI-driven, large-scale exploratory experiment that mined the entire Europe PMC database (~7 million publications) via the EPMC API using the open-weight GPT-OSS-120B model (120 billion parameters; see Supplementary Fig. 3; all codes, prompts, and intermediate data files generated during the literature search and subsequent analyses are publicly available). GPT-OSS-120B is a reasoning-capable model with a configurable reasoning-effort parameter. Feature extraction was run at the default reasoning-effort setting, while factor categorisation and deduplication were run with reasoning_effort set to 'medium'. Only the model’s final message content was retained for downstream processing. Intermediate chain-of-thought was not stored or used in any downstream analysis.

In the first step, we conducted a keyword-based search to retrieve all publications reporting associations between the mental health-related variables of interest and contextual features potentially identifiable by VLMs. We used *Psychology* as a mandatory keyword, complemented by optional terms capturing mental health-related variables (i.e. positive affect, negative affect, stress) and contextual features (e.g. environmental, social, biological or living elements). This search yielded 64,597 publications. In the second step, the open-weight GPT-OSS-120B model (temperature = 0.1) automatically extracted and structured relevant information from these publications, yielding 43,263 contextual features reported across 17,380 publications. In the third step, the same LLM (temperature = 0) condensed these multiword contextual features into concise one- to two-word categories suitable for VLM-based assessment.

In the fourth step, after removing non-responses, seven datasets were generated, capturing all combinations of increases and decreases in positive and negative affect and stress, including one dataset containing contextual features for which studies had examined one or more mental health-related variables but reported no significant associations. In the fifth step, the LLM (temperature = 0) was used to cluster conceptually similar categories within each of these seven datasets into broader groups, resulting in 6559 contextual features. After excluding those contextual features without significant associations, which would not allow meaningful replication within our dataset, 6384 contextual features remained. In the sixth step, three separate datasets were created, one each for positive affect, negative affect and stress, combining all findings on increases and decreases and removing duplicates, retaining only those contextual features for which at least three independent studies reported associations with increases or decreases in the respective mental health-related variables, yielding 1123 feature-outcome associations. After merging across the three mental health-related variables and removing duplicates, a final set of 997 unique contextual features remained, which served as the basis for the statistical analyses (for a full list of detected features for each health-relevant variable and expected effect direction, see GitHub repository).

The subsequent VLM-based ratings of the identified 997 contextual features followed the same general procedure as the greenness analyses using the open-weight LLaMA 4 VLM (model: Maverick-17B-128E-Instruct-FP8; ≈17 billion active parameters; temperature = 0.6), with the exception that only a single inference run per category was conducted for computational efficiency. The 997 literature-derived features were rated using an analogous single-category prompt, following the same prompting strategy described above, but with a JSON output format:

*Analyze the image regarding the presence of the following category: '{category_name}'. Return a JSON with three fields: 'presence': 1–10 value indicating the presence (1 = absolute absence, 10 = dominance presence); 'confidence': 1–10 value indicating confidence regarding the presence of the category; 'description': a brief description of what is seen in the image. Return JSON only*.

### Statistical analyses

All statistical analyses, including descriptive, correlational and multilevel modelling procedures, were conducted in R (version 4.4.1)^32^.

Using multilevel linear models implemented in the *lme4* package (version 1.1-35.5)^33^, we examined the primary research questions of whether VLM-derived greenness indicators (greenness, nature score, plant presence, natural light exposure and inside/outside) (i) corresponded to participants’ self-reported greenness and whether these VLM-based measures were (ii) associated with mental health-relevant factors (i.e. affect). The VLM-derived greenness indicators (aggregated over five runs) served as dependent variables, and participants were included as random effects. For analysis (i), self-reported greenness was entered as the fixed predictor at Level 1 (within-person) and Level 2 (between-person). For analysis (ii), positive and negative affect were entered as fixed predictors at Level 1 (within-person) and Level 2 (between-person). In these analyses, within-person variables (state, Level 1) were person-mean centred to capture fluctuations around each individual’s average level, while between-person variables (trait, Level 2) were computed as each participant’s mean across all momentary assessments. In a complementary analysis, we examined the relationship between aggregated VLM-derived greenness indicators and self-reported chronic stress, an additional mental health-related factor, by calculating Pearson product-moment correlations.

To assess the generalizability of the approach beyond a single model architecture, we applied the identical analytic pipeline using the Qwen3 VL (model: Qwen3-VL-235B-A22B-Instruct-FP8 model; ≈22 billion parameters; temperature = 0.7).

To assess the scalability of the framework, we extended the analyses beyond greenness. Specifically, we examined associations between VLM-derived ratings across a broad range of contextual features, identified through the AI-based literature mining system and mental health-related psychological variables, using the same statistical approach as described above. To illustrate scalability, we computed these analyses for each single feature out of the 1123 identified effects (described by 997 unique categories). We calculated separate analyses for positive affect (133 positive effects, 28 negative effects), negative affect (425 positive effects, 257 negative effects) and perceived stress (178 positive effects, 102 negative effects). A percentage rate of significant findings higher than 5% would argue for a detection rate of expected effects which is higher than chance.

## Supplementary information

Supplementary Data 1

Supplementary Information

Supplementary_Data_2_Figure_2_Source_Data

Supplementary_Data_2_Figure_3_Source_Data

## Data Availability

The data supporting the findings of this study are available as follows. The VLM-derived ratings used for the statistical analyses, together with the anonymized participant-level data (psychological variables), are openly available on the Open Science Framework (OSF) at https://doi.org/10.17605/OSF.IO/JM5BA^34^. The raw outputs generated by the LLM-based literature-mining pipeline are available on GitHub and archived on Zenodo at https://doi.org/10.5281/zenodo.20657504^35^. These data are publicly accessible without restriction. The original participant-generated photographs cannot be shared publicly. As first-person images of everyday environments, they may contain identifiable individuals and sensitive contextual information. Sharing them would violate the privacy and personality rights of participants and third parties. Requests for access will be handled by the corresponding author (Magdalena Wekenborg, Magdalena.wekenborg@tu-dresden.de) and answered within approximately 8 weeks. The source data underlying Figs. 2 and 3 are provided as Supplementary Data 2 and 3, respectively, and are also available on OSF at https://doi.org/10.17605/OSF.IO/JM5BA^34^. Figure 1 is a schematic representation and does not contain underlying numerical source data.

## Code availability

All analyses were performed using publicly available software. All VLM prompts and source code, including the LLM-based literature-mining pipeline, are available on GitHub (https://github.com/WekenborgLab/AmbuVision) and archived with a permanent identifier on Zenodo (https://doi.org/10.5281/zenodo.20657504^35^). The complete R code for replication of all data analyses and figures, the VLM-derived ratings used for statistical modelling, and the anonymized participant-level data are available on OSF (https://doi.org/10.17605/OSF.IO/JM5BA^34^). Details of the software versions and the specific parameters used to generate and analyse the data (e.g. model versions, temperature settings, image-processing parameters and R package versions) are provided in the 'Methods'. All code is released under the MIT License, permitting reuse and adaptation. No restrictions apply to code access.
