# Supplementary material for: Quantifying the human visual exposome with vision language models
Source: Commun AI Comput. 2026 Nov 5;1(1):25. doi: 10.1038/s44488-026-00023-x (PMC13739718; doi:10.1038/s44488-026-00023-x)
Supplement: Supplementary file 1 [file 44488_2026_23_MOESM1_ESM.pdf]

Supplementary Information – Overview

This document contains the following supplementary materials:

Supplementary Tables

- **Supplementary Table 1.** Original and mean values of VLM-derived greenness-related image metrics across five runs for LLaMA 4 VLM and Qwen3 VL.
- **Supplementary Table 2.** Multilevel models predicting greenness and plant presence derived from the simpler baseline methods (green-pixel count and object detector) via health-related factors.
- **Supplementary Table 3.** Multilevel models predicting Qwen3 VL-derived greenness indicators via health-related factors.

Supplementary Figures

- **Supplementary Figure 1.** Associations between chronic stress and greenness/plant-presence estimates derived from the simpler baseline methods.
- **Supplementary Figure 2.** Associations between chronic stress and Qwen3 VL-derived greenness estimates.
- **Supplementary Figure 3.** AI-driven workflow of the literature-derived visual exposome.

Supplementary Methods

- Detailed description of the two computationally cheaper baseline methods (green-pixel count and YOLOv8x-OIV7 object detection) used to benchmark the VLM-derived greenness and plant-presence ratings.

Supplementary Table 1. Original and mean values of VLM-greenness-related image metrics over 5 runs for LLaMA4 VLM and Qwen3 VL

	**Greenness**			**Natural light exposure**			**Plant presence**			**Nature score**			**Inside/outside**			
	M	SD	Min-max	M	SD	Min-max	M	SD	Min-max	M	SD	Min-max	M	SD	Min-max	
Run 1	3.15	2.86	1-10	4.88	3.36	1-10	3.45	3.29	1-10	2.77	2.61	1-9	1.25	0.43	1-2	Llama 4 VLM
Run 2	3.13	2.84	1-10	4.85	3.36	1-10	3.45	3.30	1-10	2.75	2.59	1-9	1.25	0.43	1-2	
Run 3	3.14	2.85	1-10	4.88	3.37	1-10	3.45	3.30	1-10	2.75	2.59	1-9	1.25	0.43	1-2	
Run 4	3.13	2.85	1-10	4.86	3.36	1-10	3.45	3.29	1-10	2.74	2.59	1-9	1.24	0.43	1-2	
Run 5	3.13	2.86	1-10	4.84	3.37	1-10	3.44	3.30	1-10	2.75	2.60	1-10	1.25	0.43	1-2	
Mean	3.13	2.83	1-10	4.86	3.19	1-10	3.45	3.26	1-10	2.75	2.54	1-9	1.25	0.41	1-2	
Mean confidence	9.13	1.04	2.2-10	8.84	1.02	2.8-10	9.44	0.85	2.8-10	9.02	0.90	6-10	8.15	0.54	1-10	
Run 1	3.61	2.93	1-10	6.16	2.29	1-10	3.76	3.40	1-10	3.23	2.64	1-10	1.20	0.40	1-2	Qwen3 VL
Run 2	3.61	2.93	1-10	6.17	2.31	1-10	3.76	3.41	1-10	3.24	2.64	1-10	1.20	0.40	1-2	
Run 3	3.61	2.93	1-10	6.18	2.28	1-10	3.78	3.40	1-10	3.23	2.64	1-10	1.20	0.40	1-2	
Run 4	3.61	2.93	1-10	6.18	2.29	1-10	3.76	3.40	1-10	3.23	2.64	1-10	1.20	0.40	1-2	
Run 5	3.61	2.93	1-10	6.17	2.31	1-10	3.76	3.39	1-10	3.23	2.64	1-10	1.20	0.40	1-2	

Mean	3.61	2.92	1-10	6.17	2.27	1-10	3.76	3.39	1-10	3.23	2.63	1-10	1.20	0.40	1-2
Mean confidence	9.13	0.95	3-10	8.47	0.82	4-10	9.47	0.77	3.6-10	9.01	0.92	4-10	9.79	0.43	5-10

Supplementary Table 2. Multilevel model predicting categories of greenness derived from cheaper methods (green-pixel counter and a standard object detector) via health-related factors.

	**Greenness**			**Plant presence**		
Predictors	*Estimates*	*Conf. Int (95%)*	*p-value*	*Estimates*	*Conf. Int (95%)*	*p-value*
Intercept	0.45	-0.32 – 1.22	0.248	0.21	-0.29 – 0.70	0.416
Positive affect trait (Level 2)	0.24	0.04 – 0.44	**0.018**	0.12	-0.00 – 0.25	0.051
Positive affect state (Level 1)	0.10	0.04 – 0.16	**0.001**	0.03	-0.03 – 0.09	0.390
Negative affect trait (Level 2)	-0.44	-0.79 – -0.10	**0.011**	-0.15	-0.37 – 0.07	0.188
Negative affect state (Level 1)	-0.03	-0.13 – 0.08	0.594	0.05	-0.04 – 0.15	0.283
**Random Effects**						
σ^2	1.12			1.01		
τ_{00}	0.26 $_{Participant}$			0.08 $_{Participant}$		
ICC	0.19			0.07		
N	106 $_{Participant}$			106 $_{Participant}$		
Observations	2671			2671		
Marginal R^2 / Conditional R^2	0.031 / 0.215			0.007 / 0.079		

Note. Statistical significance for fixed effects was determined using two-sided Wald t-tests within a linear mixed-effects framework. Exact p-values are provided for all fixed effects in the table. No adjustments were made for multiple comparisons. Level 1 variables represent state-level observations; Level 2 variables represent participant-level traits. Confidence intervals (Conf. Int.) are reported at the 95% level. σ^2 = residual variance; τ_{00} = random intercept variance; ICC = Intraclass Correlation Coefficient.

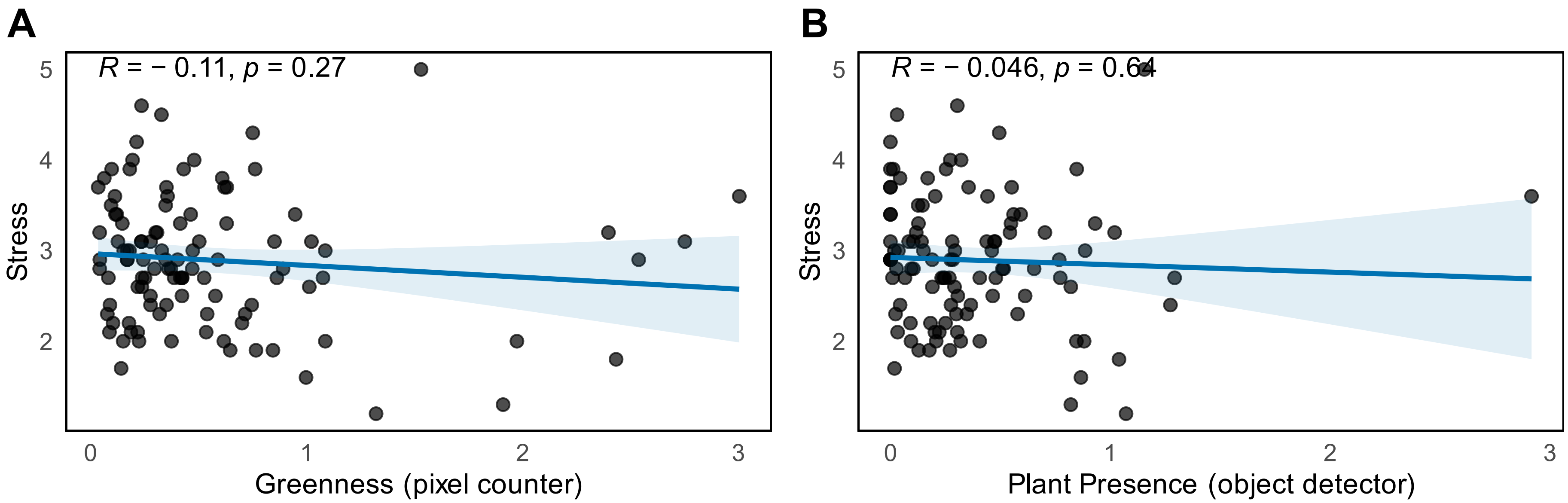

Supplementary Fig.1: Greenness-stress associations based on greenness estimates derived from cheaper methods. Trait-level associations between greenness estimates (by green-pixel counter [A] and a standard object detector [B]) and chronic stress. Higher greenness and plant presence were not associated with lower chronic stress.

Supplementary Table 3. Multilevel model predicting VLM-derived categories of greenness from the Qwen3 VL model via health-related factors.

	**Greenness**			**Natural light exposure**			**Plant presence**			**Nature score**			**Inside/outside**			**Average score**		
Predictors	*Estimates*	*Conf. Int (95%)*	*p-value*	*Estimates*	*Conf. Int (95%)*	*p-value*	*Estimates*	*Conf. Int (95%)*	*p-value*	*Estimates*	*Conf. Int (95%)*	*p-value*	*Estimates*	*Conf. Int (95%)*	*p-value*	*Estimates*	*Conf. Int (95%)*	*p-value*
Intercept	3.30	1.37 – 5.24	**0.001**	5.24	3.97 – 6.52	**<0.001**	3.18	0.87 – 5.50	**0.007**	2.54	0.75 – 4.33	**0.005**	1.23	1.03 – 1.43	**<0.001**	3.57	1.89 – 5.25	**<0.001**
Positive affect trait (Level 2)	0.60	0.10 – 1.10	**0.018**	0.44	0.11 – 0.77	**0.008**	0.64	0.04 – 1.24	**0.035**	0.65	0.19 – 1.12	**0.006**	0.02	-0.03 – 0.07	0.412	0.59	0.15 – 1.02	**0.008**
Positive affect state (Level 1)	0.10	-0.05 – 0.25	0.193	0.19	0.07 – 0.32	**0.002**	-0.01	-0.18 – 0.17	0.945	0.05	-0.08 – 0.19	0.434	0.07	0.04 – 0.09	**<0.001**	0.09	-0.05 – 0.22	0.201
Negative affect trait (Level 2)	-1.06	-1.92 – -0.20	**0.016**	-0.23	-0.79 – 0.33	0.424	-0.91	-1.94 – 0.12	0.082	-0.87	-1.66 – -0.07	**0.032**	-0.07	-0.15 – 0.02	0.141	-0.77	-1.51 – -0.02	**0.044**
Negative affect state (Level 1)	0.06	-0.19 – 0.31	0.650	-0.04	-0.24 – 0.17	0.725	0.09	-0.21 – 0.38	0.559	0.02	-0.20 – 0.25	0.841	-0.02	-0.06 – 0.01	0.208	0.03	-0.19 – 0.25	0.767

**Random Effects**						
σ^2	6.64	4.43	8.97	5.30	0.14	4.94
τ_{00}	1.68 $_{Participant}$	0.64 $_{Participant}$	2.43 $_{Participant}$	1.46 $_{Participant}$	0.01 $_{Participant}$	1.27 $_{Participant}$
ICC	0.20	0.13	0.21	0.22	0.08	0.20
N	106 $_{Participant}$	106 $_{Participant}$	106 $_{Participant}$	106 $_{Participant}$	106 $_{Participant}$	106 $_{Participant}$
Observations	2671	2671	2671	2671	2671	2671
Marginal R^2 / Conditional R^2	0.029 / 0.225	0.016 / 0.140	0.020 / 0.229	0.032 / 0.241	0.015 / 0.098	0.028 / 0.226

Note. Statistical significance for fixed effects was determined using two-sided Wald t-tests within a linear mixed-effects framework. Exact p-values are provided for all fixed effects in the table. No adjustments were made for multiple comparisons. Level 1 variables represent state-level observations; Level 2 variables represent participant-level traits. Confidence intervals (Conf. Int.) are reported at the 95% level. σ^2 = residual variance; τ_{00} = random intercept variance; ICC = Intraclass Correlation Coefficient.

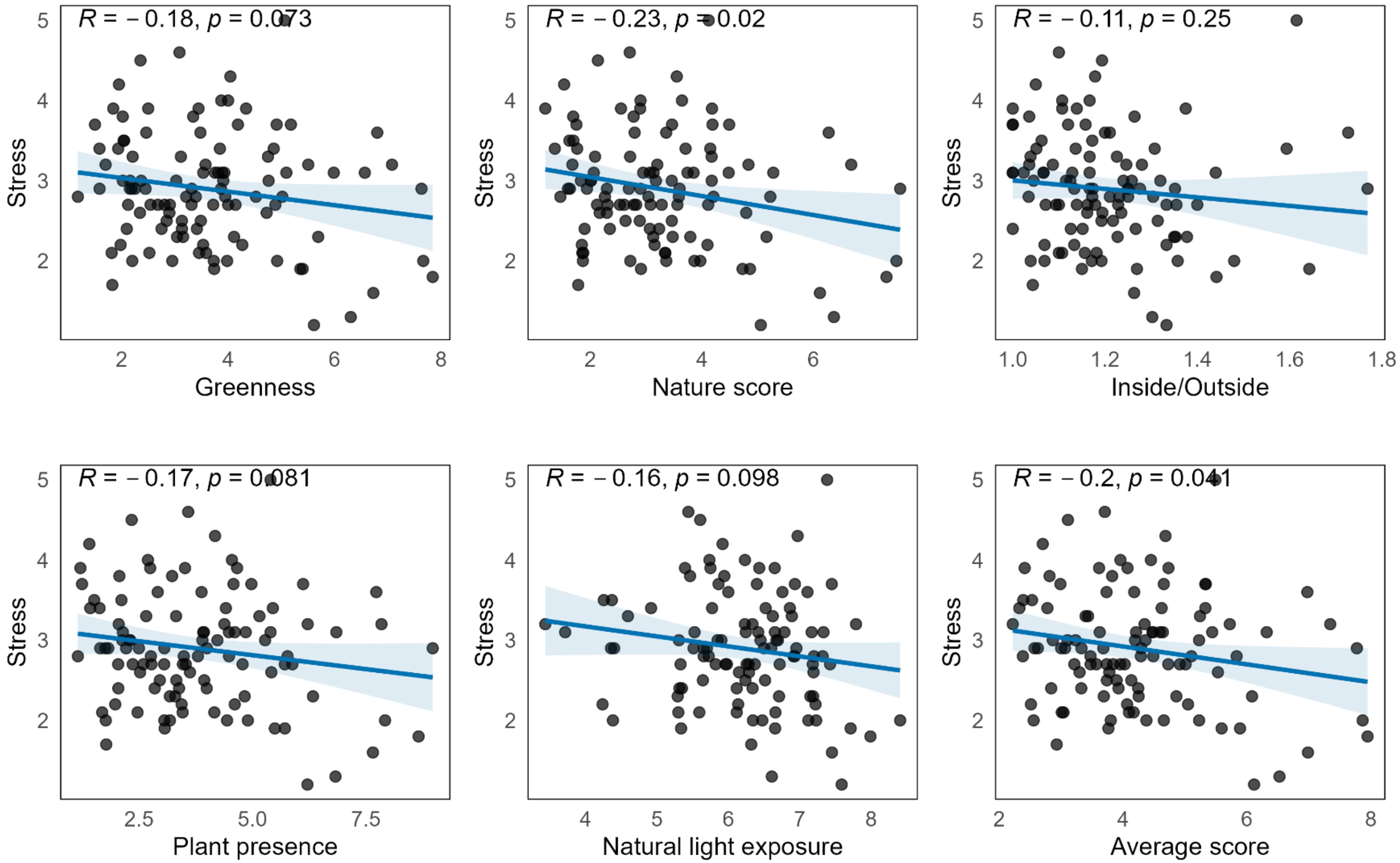

Supplementary Fig.2: Greenness-stress associations based on greenness estimates derived from Qwen3 VL. Trait-level associations between Qwen3 VL-derived greenness estimates and chronic stress. Higher greenness was associated with lower chronic stress, with all greenness indicators showing negative correlations of comparable magnitude.

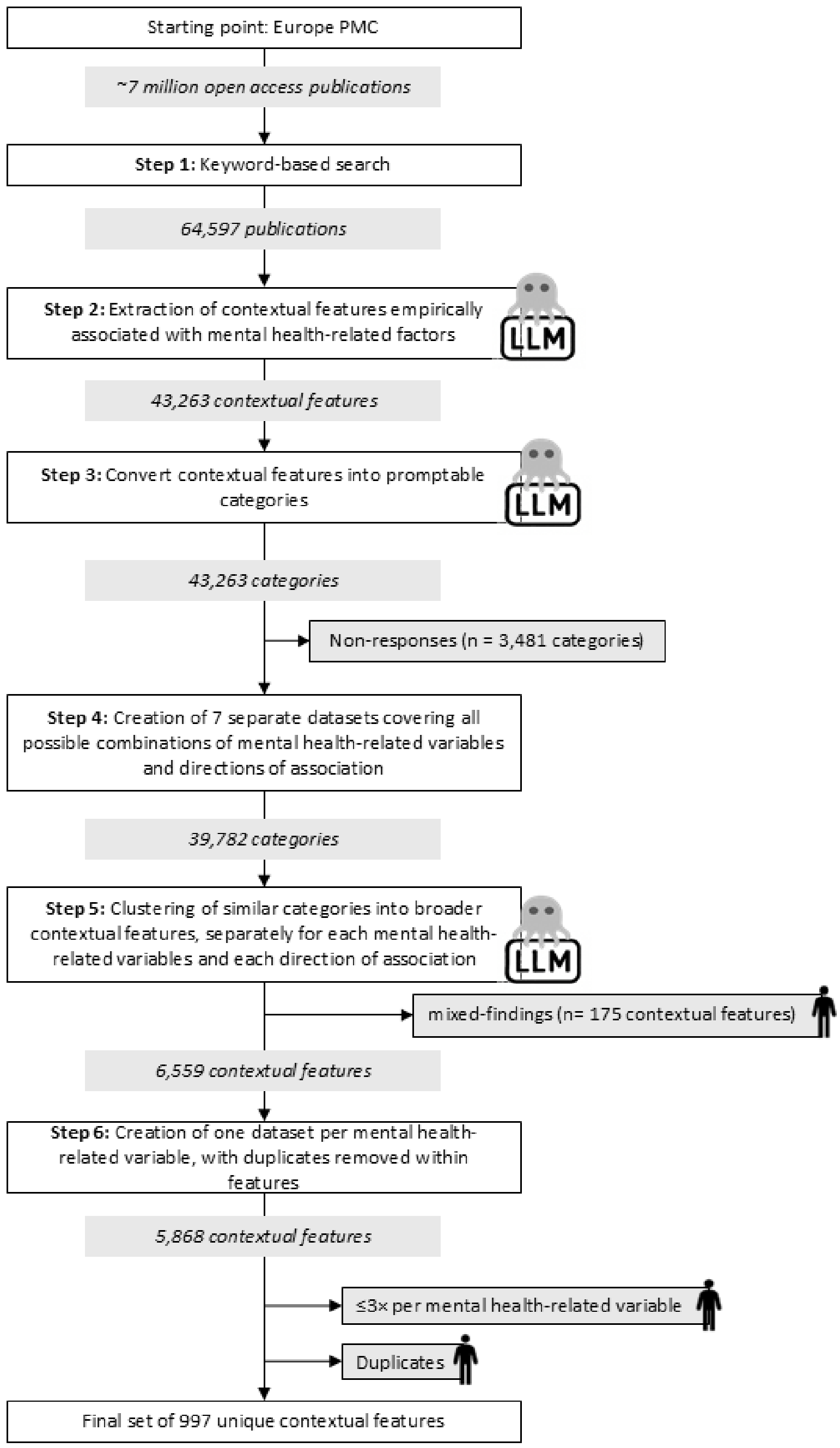

Supplementary Fig.3. AI-Driven Workflow of the Literature-Derived Visual Exposome. The figure illustrates the AI-driven workflow used to extract and organize environmental contextual features from the scientific literature. A large language model (LLM) processes millions of full-text publications to identify variables relevant to mental health and group them into coherent categories that collectively define the literature-derived visual exposome used in the present study. Pictograms of a person indicate human-in-the-loop steps involving targeted manual oversight or refinement.

Supplementary Methods

Detecting Greenness and Plant presences by means of computational cheaper methods not using VLMs.

Green-pixel-count method

Each photograph was converted from RGB to the HSV color space using the PIL convention, in which H, S, and V each take values in [0, 255]. A pixel was classified as "green" if it satisfied all three thresholds simultaneously:

- Hue: $50 \leq H \leq 114$, corresponding to approximately 70°–160° on the standard 0°–360° hue circle (i.e., yellow-green through blue-green);
- Saturation: $S \geq 40$ (~16%), excluding near-grey pixels;
- Value: $V \geq 30$ (~12%), excluding near-black pixels.

The HSV thresholds ($H \in [70°, 160°]$; $S \geq 16\%$; $V \geq 12\%$) are not tunable parameters of the model. The hue range corresponds to the green sector of the HSV cylinder (i.e., the half-circle around the standard green primary at 120°, bounded short of yellow [60°] and cyan [180°]). The saturation and value floors exclude pixels for which hue is mathematically undefined or numerically unstable (near-grey and near-black, respectively); their precise values are inconsequential within roughly an order of magnitude. The thresholds were fixed before any greenness score was computed and before any association with affect or stress was examined, ruling out outcome-based tuning. The fraction of pixels satisfying the rule (green_pixel_frac_hsv ∈ [0, 1]) was multiplied by 10 to yield a greenness score on a scale from 0 to 10.

Object-detection method

YOLOv8x-OIV7 (Ultralytics, ~68 M parameters) is a closed-vocabulary detector pretrained on Open Images V7 (600 classes); detections were filtered to the single class Plant (OIV7 index 393) at a confidence threshold of 0.10 and 1280-pixel input. The per-image coverage was computed as the union of axis-aligned plant bounding boxes, rasterized onto a 256×256 grid to avoid double-counting overlapping detections, divided by image area. The final score is PlantPresence = round(coverage × 10, 2), mapping directly onto the 0 to 10 scale used for the VLM derived PlantPresence score. Inference was run on a single NVIDIA Quadro RTX 8000.
