# Supplementary material for: Quantifying the human visual exposome with vision language models
Source: Commun AI Comput. 2026 Nov 5;1(1):25. doi: 10.1038/s44488-026-00023-x (PMC13739718; doi:10.1038/s44488-026-00023-x)
Supplement: Supplementary file 2 [file 44488_2026_23_MOESM2_ESM.pdf]

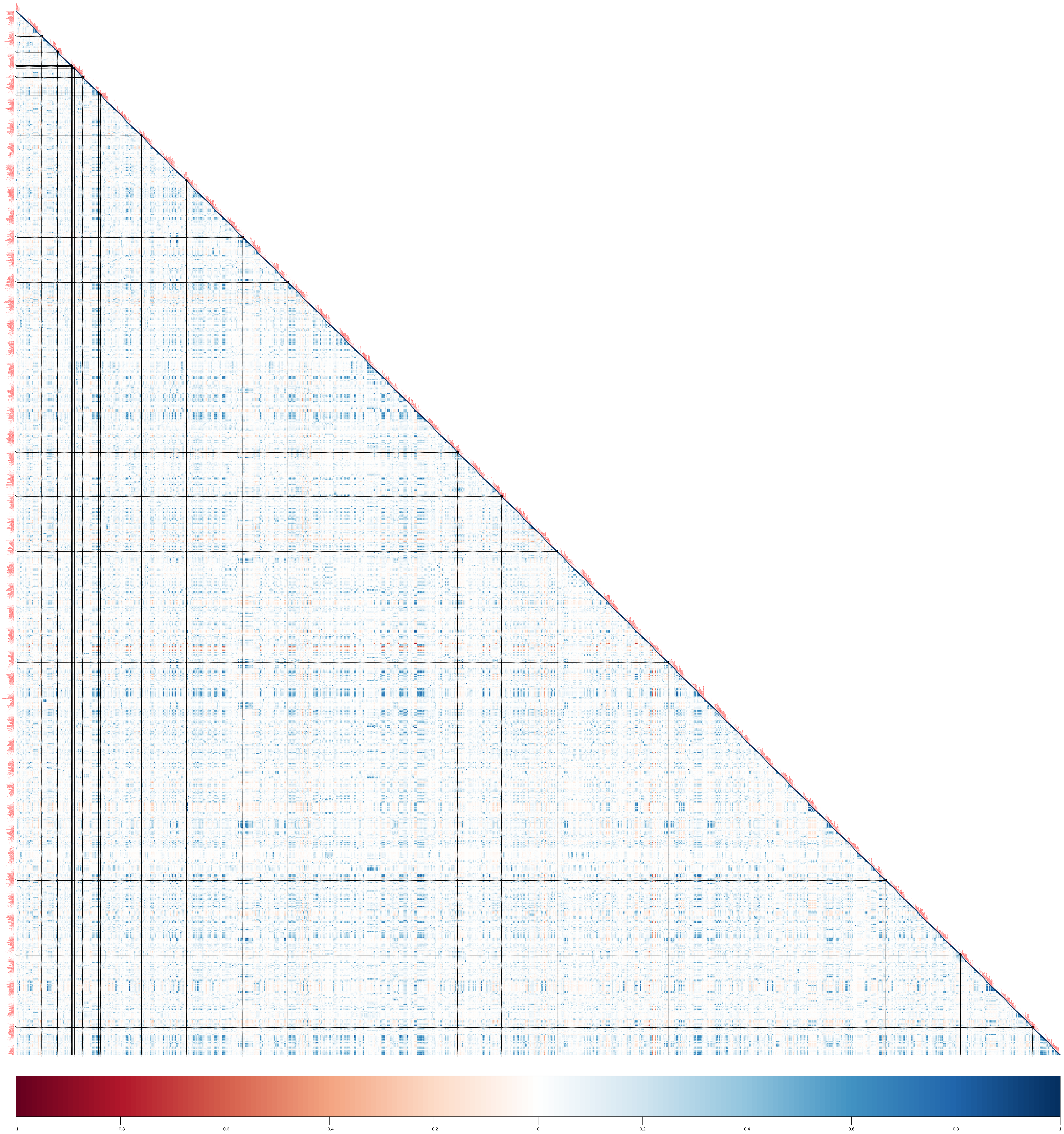

−1
−0.8
−0.6
−0.4
−0.2
0
0.2
0.4
0.6
0.8
1
